# Effectiveness of Perioperative Auricular Therapy on Postoperative Pain after Total Hip Arthroplasty: A Systematic Review and Meta-Analysis of Randomised Controlled Trials

**DOI:** 10.1155/2019/2979780

**Published:** 2019-03-03

**Authors:** Xin-Xin Ye, Yu-Zhen Gao, Zhong-Bo Xu, Qi-Xi Liu, Chen-Ju Zhan

**Affiliations:** ^1^Department of Nursing, Mindong Hospital of Ningde City, Fuan, Fujian 355000, China; ^2^School of Nursing, Fujian University of Traditional Chinese Medicine, Fuzhou, Fujian 350122, China; ^3^Department of Molecular Diagnosis, Clinical Medical School, Yangzhou University, Subei People's Hospital, Yangzhou, Jiangsu 225000, China; ^4^Emergency Department, Affiliated Hospital of Jiangxi University of Traditional Chinese Medicine, Nanchang, Jiangxi 330006, China

## Abstract

Auricular therapy (AT) is a conventional therapy in traditional Chinese medicine. However, the effectiveness of perioperative AT in pain treatment after total hip arthroplasty (THA) is still controversial. Nine randomised controlled trials (RCTs) involving 605 patients who have undergone THA with or without AT from inception to March 2018 were collected and included in this study by searching more than 12 databases (e.g., PubMed, Excerpta Medica Database, and Cochrane Library). A random-effects model that pooled seven articles showed that the visual analogue scale (VAS) in the AT group was lower than that of the control group at each postoperative time point in patients after THA, except at the time points of 6 and 36 h. The intraoperative body mass-adjusted fentanyl amount in the AT group was also lower than that of the control group in two trials. The other outcomes (time to first analgesic request and incidence of postoperative nausea and vomiting, perioperative bradycardia, and transitory hypotension) showed insignificant difference. Then, subgroup analysis showed similar results to those of the total articles with the term “VAS”. Regression analysis found that the prolonged time after the operation decreased the difference in VAS between the two groups. Although all the outcomes were assessed as very low to low in the GRADE system, evidence on the effectiveness of perioperative AT in pain treatment after total hip replacement was positive.

## 1. Introduction

For patients who have hip joint disease, total hip arthroplasty (THA) is one of the most successful treatments for end-stage osteoarthritis and other hip diseases [1]. Generally, THA is an effective approach to relieve pain and improve function and quality of life for various hip diseases [2, 3]. Over the past decade, the incidence of THA has increased both in elderly and young individuals, and this number is estimated to reach 572,000 cases by 2030 [4, 5]. The treatments of THA can cause severe perioperative pain in patients [6]. Almost all postoperative pain is caused by tissue damage at the site of the operation. Postoperative wound pain is the most serious problem after surgery [7, 8]. In an early review, the risk of persistent pain after surgery has been described [9]. One out of three patients with THA experience moderate to severe pain during movement. Hence, medical workers and patients face the challenge of postoperative pain [10]. Postoperative pain not only hinders new exercise and rehabilitation but also affects overall recovery [11]. Acute postoperative pain management via opioid analgesics was also lower than the optimal for patients with total joint replacement because side effects, such as nausea, vomiting and, in particular, sedation, can interfere with rehabilitation [12, 13].

Complementary and alternative medicine is readily accepted by both developing and developed countries, where it is increasingly regarded as a substitute for conventional therapies and recommended to reduce the postoperative pain [14–16]. AT is defined as a method of acupuncture wherein the external surface of the ear or auricle is stimulated to alleviate pathological conditions in other parts of the body [17]. In the future, AT may become a type of therapy in multimodal pain management protocols. AT that is generally carried out by medical professionals in some Eastern countries is easy to operate, economical and a safe therapy for patients. In 1982, the World Health Organisation set up a working group to standardise the research and clinical applications of AT [18]. Auricular acupuncture and homoeopathic arnica have clinically desirable effects of decreasing analgesic administration and postoperative swelling [19].

A systematic review has indicated that acupuncture may be a feasible adjuvant therapy for pain after total hip or knee arthroplasty [20] because it can relieve postoperative pain and reduce the dosage of opioid analgesics and other related side effects [21]. However, the previous study did not provide strong evidence to prove that AT is an effective analgesic method in relieving postoperative pain [22]. Our meta-analysis may provide an objective theoretical basis for clinical decision-making to evaluate the clinical efficacy of AT on postoperative pain for patients after THA.

## 2. Materials and Methods

### 2.1. Search Methods

Electronic searching and citation snowballing were both used to locate relevant studies. A total of 12 electronic databases, including PubMed, Excerpta Medica Database (Embase), Cochrane Central Register of Controlled Trials, Web of Science, Science Direct, PsycINFO, Cumulative Index to Nursing and Allied Health Literature, Allied and Complementary Medicine, China National Knowledge Infrastructure, Chinese Biomedical Literature Database (CBM), WanFang, and Chinese Scientific Journal Database, were searched for relevant studies that were conducted from inception to March 2018. The search strategy of all available databases was determined by the principle of “PICOS” and its details were presented in the* Supplemental Materials* (available here). For the related inventions and patients, we used “auriculotherapy”, “acupuncture”, “ear”, “arthroplasty”, “replacement”, “hip”, and “total hip arthroplasty” as MeSH terms. For the outcomes, “pain” and “postoperative” were the MeSH terms we used.

### 2.2. Inclusion and Exclusion Criteria

Studies were considered eligible if they met the following criteria: (1) randomised controlled trials (RCTs) as the design type of studies; (2) patients who underwent THA regardless of gender and disease types; (3) the intervention treatment limited to AT (AT includes auricular acupuncture, auricular point buried-bean, auricular massage, auricular magnetic therapy, and auricular moxibustion); patients undergoing AT with or without conventional treatments can be regarded as the same type in our meta-analysis; (4) patients who underwent conventional treatment with or without sham–AT regarded as the control group. Clinical animal trials, case reports, and nonrandomised controlled trials were excluded.

### 2.3. Data Extraction and Quality Assessment

Two reviewers (Ye XX and Gao YZ) independently screened the titles and abstracts of all articles and excluded those unrelated to the specified selection criteria. The data were extracted independently into a sheet that included a prespecified set of variables (articles' general information, including author names; publication year and country; patient characteristics, such as sample size, mean age, and disease types; interventions groups; main outcomes with VAS; and any other relevant findings). Data were also extracted from any author in the collaboration group. The Cochrane Collaboration Handbook Tool for the systematic reviews of interventions was used to estimate the risk of bias for each article, including random sequence generation, allocation concealment, blinding of participants and personnel, blinding of outcome assessment, incomplete outcome data and selective reporting and other biases [32]. Any disagreement between the investigators was resolved by obtaining a consensus among the full review team.

### 2.4. Process of Auricular Therapy

The steps for the AT process or APBB by the acupuncturist are as follows: (1) selecting the specific auricular acupoints, (2) sterilising the auricular acupoints, (3) embedding the needles or vaccaria seeds (a kind of plant seed or bean) in the auricular acupoints, (4) fixing the needles or vaccaria seeds with medical adhesive tape, and (5) pressing the auricular acupoints according to patients' needs. Last, the retention time of needles or vaccaria seeds would keep in one to seven days in the process of the auricular therapy [24, 25].

### 2.5. Types of Outcome Measures

The primary outcome measured by VAS in our meta-analysis was postoperative pain. The secondary outcomes were body mass-adjusted fentanyl amount (*μ*g/kg) in intraoperative time, postoperative time to first analgesic request (min) and perioperative complications (postoperative nausea and vomiting (PONV), perioperative transitory hypotension and bradycardia).

### 2.6. Statistical Analysis

The heterogeneity of the included studies was assessed using Q statistics and I^2^ index according to the suggestions of the Cochrane Collaboration.* P* < 0.05 with I^2^ index > 50% was considered to show significant heterogeneity. The estimates (standardised mean difference (SMD) or odds ratio (OR)) with 95% confidence interval (CI) were pooled with a fixed-effects model if the heterogeneity was significant. Otherwise, the estimates were pooled with a random-effects model that accounted for both within- and between-study variability. We also conducted subgroup analysis and metaregression by using the variables time group, GRADE quality, control intervention type in the control group, starting point of treatment, and other related variables to assess the impacts on outcomes. All analyses were performed using RevMan version 5.3 from the Cochrane website or STATA version 14.0 (StataCorp, College Station, Texas). P < 0.05 was considered statistically significant except otherwise specified.

### 2.7. GRADE Quality of Metaevidence

GRADE guidance tools, including nine RCTs, were used to assess the quality of evidence for the metaresults. The GRADE framework characterises the quality of evidence on the basis of study risk of bias, publication bias, imprecision, inconsistency, and study indirectness with the levels of high, moderate, low, and extremely low for each outcome.

## 3. Results

### 3.1. Literature Search

**Figure 1:**
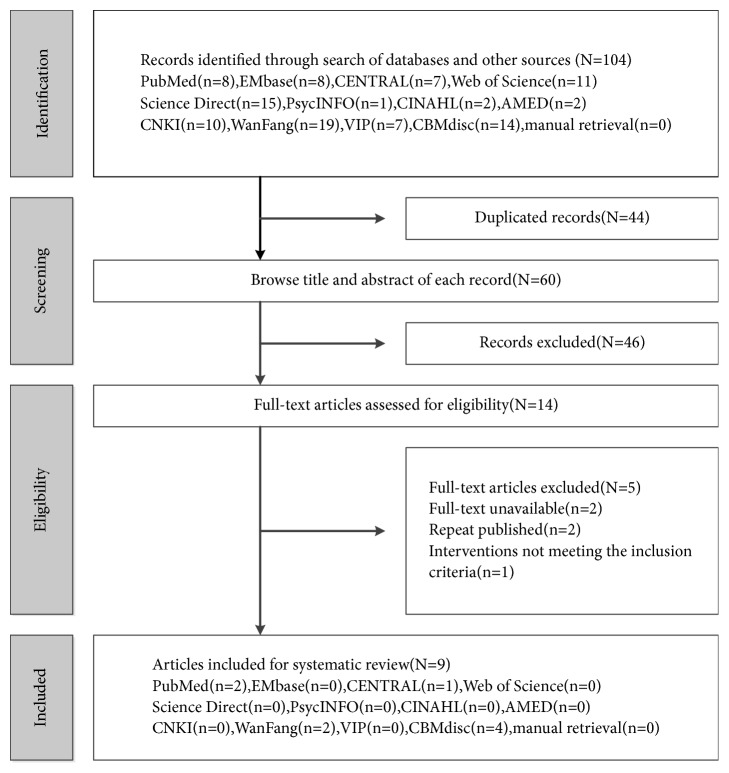
*Flowchart of study selection*. CENTRAL: Cochrane Central Register of Controlled Trials, CNKI: China National Knowledge Infrastructure, VIP: Chinese Scientific Journal Database, and CBM: Chinese Biomedical Literature Database.

A total of 104 relevant citations were identified from the database search, and 46 potentially eligible articles were retrieved for full-text review. A total of 12 articles were excluded because of repeated publication (n = 2), absence of comparison among treatments (n = 1), and unavailability of full text (n = 2). Out of the remaining 9 RCTs, 44.4% (4/9), 22.2% (2/9), 22.2% (2/9), and 11.1%(1/9) of the articles were obtained from CBM, WANGFANG, PubMed, and Cochrane Library, respectively.  Figure 1 shows the flowchart illustrating the details of the search results.

### 3.2. Study Characteristics and Quality Assessment

A total of 605 patients from 9 articles with a wide sample size (range of 30–116, mean of 67) were enrolled in our meta-analysis. Most of them were strictly RCTs. A total of 33.3% (3/9) and 66.7% (6/9) of the articles were published in Germany [23–25] and China [26–31], respectively. The VAS, intraoperative application amount of fentanyl, time to the first analgesic request, nausea and vomiting, perioperative bradycardia, perioperative hypotension from 77.8% (7/9) [24, 26–31], 22.2% (2/7) [23, 25], 33.3% (3/9) [23, 24, 30], 33.3% (3/9) [23, 24, 29], 11.1% (1/9) and 11.1% (1/9) of articles [23, 25] were the outcomes in our meta-analysis, respectively. The details are shown in Table 1. On top of that, auricular acupuncture and other auricular point buried-bean were used in 33.3% (3/9) [23–25] and 66.7% (6/9) [26–31] of the articles, respectively. Intraoperative general anaesthesia (GA) was performed in 66.7%(6/9) of the known recorded articles. However, three other articles did not describe any type of anaesthesia. A total of 55.6% (5/9) and 44.4% (2/9) of the articles used preoperative and postoperative AT, respectively. Sham acupuncture (SA) also was found in 44.5%(4/9) of articles. For the conventional treatments, patient-controlled analgesia (PCA) and rehabilitation exercises were performed in most of the nine RCTs. What is more, nonsteroidal anti-inflammatory drugs such as ibuprofen and celecoxib were used in two articles of them. For the details of the treatments of nine RCTs, see Table 2.

**Figure 2:**
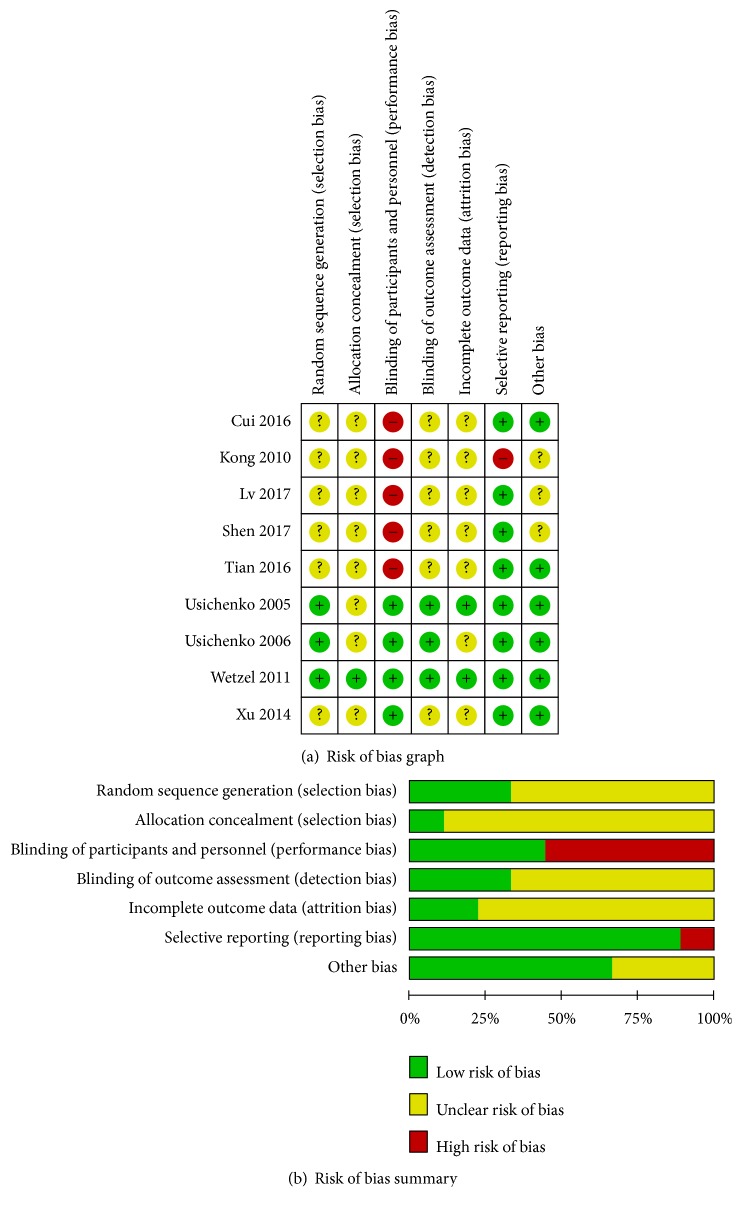
Risk of bias graph and summary of the included 9 RCTs.

The methodological quality and risk of bias for the included studies are shown in Figure 2. Among the nine RCTs, three used random number method [23–25], one exhibited appropriate allocation concealment [23], four performed participant and personnel blinding [23–25, 29], two showed incomplete outcome data [23, 24], and eight displayed selective reporting [23–26, 28–31]. The total quality of each included article was generally assessed from A to C.

### 3.3. Meta-Analysis Results

#### 3.3.1. Postoperative VAS

**Figure 3:**
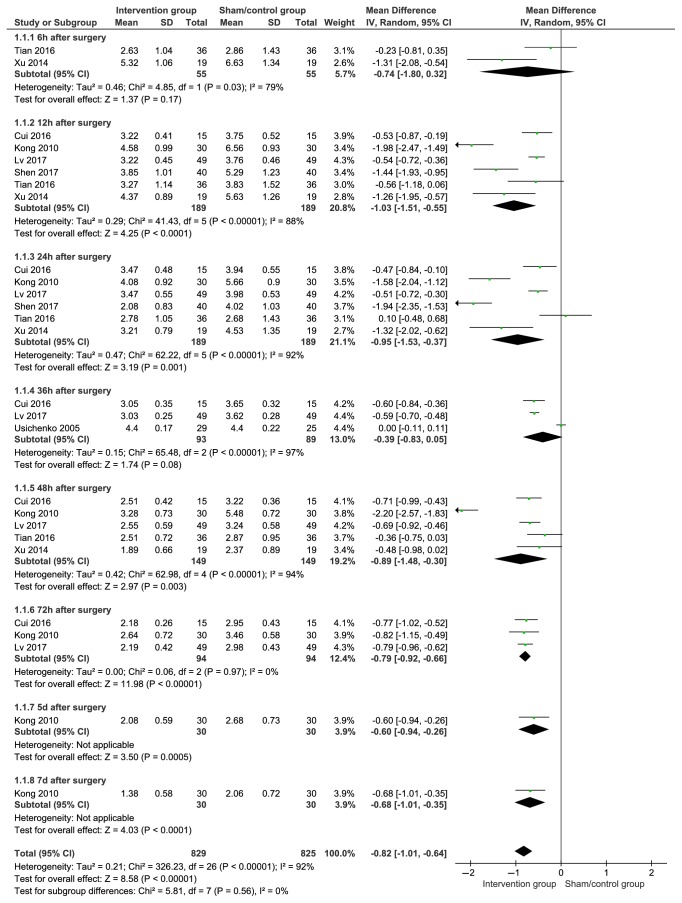
Pooled VAS pain score results in patients after auricular therapy and total hip arthroplasty.

VAS was used at different time points (postoperative 6 h, 12 h, 24 h, 36 h, 48 h, 72 h, 5 and 7 days) on patients after hip arthroplasty in the total of seven articles. The results were pooled using a randomised effect model because of the high heterogeneity. On the subgroup analysis, the observation time points of postoperative 12 h (SMD with 95%CI=−1.03(−1.51,−0.55), P<0.001), postoperative 24 h (SMD with 95%CI=−0.95 (−1.53, −0.37), P=0.001), P=0.08), postoperative 48 h (SMD with 95%CI=−0.89 (−1.48, −0.30), P=0.003), postoperative 72 h (SMD with 95%CI=−0.79 (−0.92, −0.66), P<0.001), postoperative 5 days (SMD with 95%CI=−0.60 (−0.94, −0.26), P<0.001), and postoperative 7 days (SMD with 95%CI=−0.68 (−1.01, −0.35), P<0.001) were found such that the pooled results of VAS of the AT group were lower than that of the control group, but not the time points of postoperative 6 h (SMD with 95%CI=−0.74 (−1.80, 0.32), P=0.17) and postoperative 36 h (SMD with 95%CI=−0.39 (−0.83, 0.05). The details are shown in Figure 3.

#### 3.3.2. Body Mass-Adjusted Fentanyl Amount and Time to First Analgesic Request

**Figure 4:**
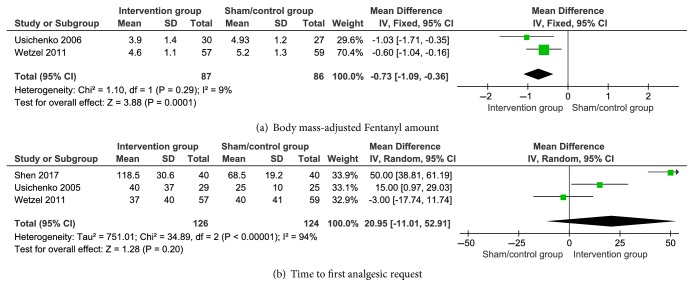
Pooled body mass-adjusted Fentanyl amount and time to first analgesic request results in the patients after auricular therapy and total hip arthroplasty.

Body mass-adjusted fentanyl amount was pooled using a fixed-effects model with low heterogeneity (P = 0.29, I^2^ = 9%).  Figure 4 shows that the experimental group had lower values than the control group (SMD with 95% CI = −0.73 (−1.09, −0.36), P = 0.0001). The time to first analgesic request also showed insignificant difference between the two groups (SMD with 95% CI = 20.95 (−11.01, 52.91), P = 0.20). The details are shown in Figure 4.

#### 3.3.3. Perioperative Complications

**Figure 5:**
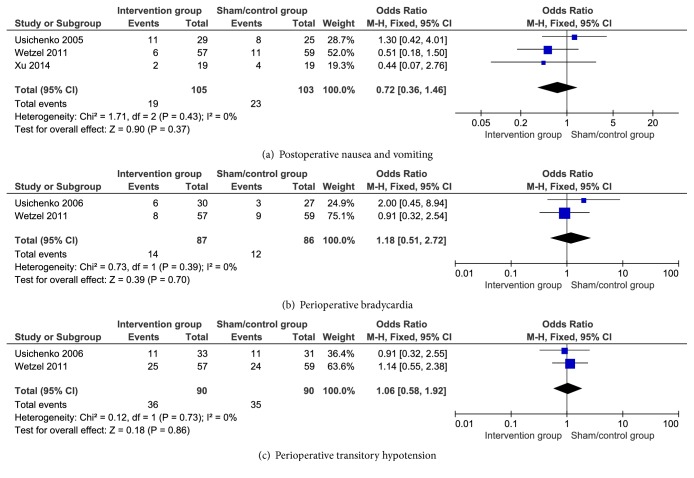
Postoperative nausea and vomiting, perioperative transitory hypotension, and bradycardia results in the patients after auricular therapy and total hip arthroplasty.

The incidences of PONV (OR, 95% CI = 0.72 (0.36, 1.46), P=0.37), perioperative bradycardia (OR, 95%CI=1.18(0.51, 2.72), P=0.70), and perioperative transitory hypotension (OR, 95% CI =1.06 (0.58, 1.92), P=0.86) that were pooled using a fixed-effects model with low heterogeneity (all I^2^ = 0%) showed insignificant difference between the two groups. The details are shown in Figure 5.

### 3.4. Subgroup and Regression Analysis

**Figure 6:**
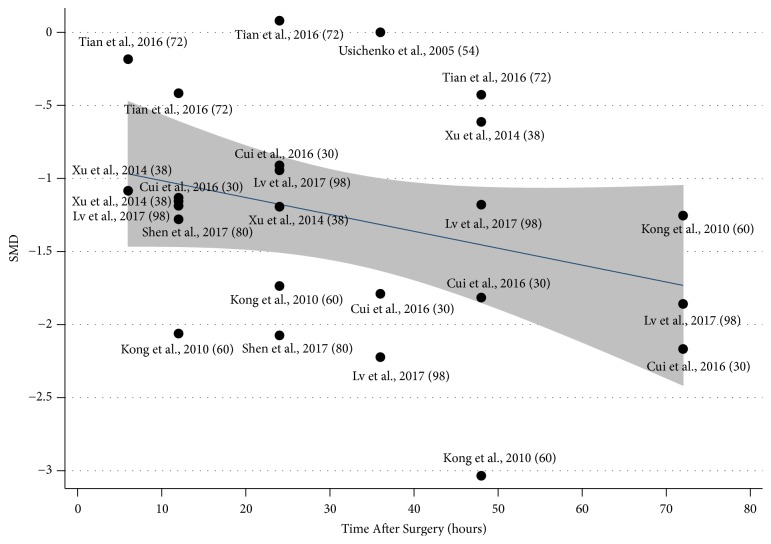
Metaregression for the VAS pain score in the patients after auricular therapy and total hip arthroplasty (P=0.108).

Most results of subgroup analysis in the different variables such as the threshold of observation time (<24h, SMD95%CI= -1.076(-1.426, -0.726), P<0.001; >24h, SMD95%CI=-1.375(-1.813, -0.938), P<0.001); <=48h, SMD95%CI=-1.174(-1.496, -0.853), P<0.001; >48h, SMD95%CI -1.394(-1.832,-0.955), P=0.021), GRADE quality (Grade C, SMD95%CI= -1.311(-1.62, -1.001), P<0.001), the type of intraoperative anaesthesia (intraoperative GA SMD95%CI= -1.111(-1.479,-0.743), P<0.001), the type of control treatment (SA+CT type SMD95%CI= -1.311(-1.620,-1.001), P<0.001; Just CT type, SMD95%CI=-0.780(-1.270, -0.290), P=0.022), Intraoperative General Analgesics (General NASIDs, SMD95%CI= -0.772(-0.172,-0.292), P=0.001; No general NASIDs, SMD95%CI=-1.217(-1.492,-0.941), P=0.002) and the starting time of AT (Preoperative, SMD95%CI= -1.241(-1.071,-1.207), P<0.001; Postoperative, SMD95%CI= -1.114(-1.221, -1.008), P<0.001) were consistent with the original results with full articles (total, SMD95%CI=-0.82(-1.012,-0.642), P<0.001), except in B-GRADE articles (SMD95%CI= -1.000 (-1.339,-0.660), P=0.591). The details are shown in Table 3. In addition, the trend of SMD with long time after THA declined was calculated by regression analysis without statistical significance (P=0.108), as shown in Figure 6.

### 3.5. GRADE Quality of the Main Outcomes

The GRADE tool was used to evaluate the evidence in the results that showed extremely low to low values for each main outcome. The details of this meta-analysis in terms of evidence quality are presented in Table 4.

## 4. Discussion

Gan et al. [33] concluded that patients mostly suffer from moderate or severe pain after THA. Subsequently, Guay et al. [34] believed that pain is associated with the increase in postoperative bleeding. PONV which is mainly caused by anaesthesia inhalation and opioid analgesics is also regarded as a common complication after anaesthesia [35]. The incidence of PONV after THA is in the range of 20%–83% which significantly affects postoperative quality of life [36, 37]. Postoperative pain, nausea, and vomiting lead to discomfort, decreased surgical satisfaction, and prolonged hospital stay [38]. Therefore, considering these serious postoperative problems, postoperative pain and perioperative complications should be decreased.

A total of 9 RCTs including 605 patients were included in our systematic review. Our results showed that the perioperative VAS value of the intervention group was significantly lower than that of the control group at different time points in patients after THA. The typical period of maximal postoperative pain after THA is 2–3 days [24]. Therefore, we conducted subgroup analysis using the variable of time points from 6 h to 7 days after THA. Fortunately, we achieved the same conclusion at different time points for patients after THA, except those at 6 and 36 h. The analgesic mechanism of AT was still unclear for patients after THA. The analgesic effect of AT can be blocked by opioid antagonists which can be used to explain the role of the endorphin system in the analgesic mechanism of AT [39]. Krause [40, 41] also found that AT could improve the pain threshold in the local area of the patients. The auricular point of Shen Men is the most used to generate analgesic, sedative and anti-inflammatory effects [42] and increased endorphin secretion and serotonin production, thereby suppressing the transmission of pain messages and pain perception [43]. In our study, the auricular point of Shen Men was selected in all articles, and hip was used as the secondary auricular point in all the patients after THA.

To explore other impact factors of the postoperative VAS results in our meta-analysis, we conducted other subgroup analyses according to some features of the articles in patients after THA. For the risk of bias, the statistical significance of postoperative VAS was only shown in C-level literature but not in the A- and B-level ones. Sham acupuncture is physiological and not inert [44]. It can produce the measurable clinical effects for patients by providing the analgesic effect in 40%–50% of the patients, but it obtained 60% for true acupuncture [45]. However, when we conducted subgroup analysis, we obtained consistent results for patients who underwent THA and were treated with or without sham acupuncture. Although, there was no difference between the subgroup of the starting points of the AT in whole time, we found that there was a difference between the two groups at six hours after surgery in our meta-analysis [29, 31]. It means that the different onset time of adjuvant analgesic may affect the postoperative VAS results.

We found that the body mass-adjusted fentanyl amount under intraoperative time for the patients after THA was lower than those of the AA and control groups. It is similar with the research obtained results of Wetzel et al. [25]. The difference in the required fentanyl between the two groups prompted the treatment of AA and can support the analgesic effect for patients with chronic and acute postoperative pain [46, 47]. We also explored whether AT can prolong the time to first analgesic request for the patient after THA. However, the result showed insignificant differences between the two groups (Figure 4(b)). Postoperative analgesic requirements were controlled by the medical staff and directly affected by the surgery type and patient's economic condition [46]. Thus, time to the first analgesic request is a particularly unreliable outcome in assessing the effects of AT.

Multimodal pain management protocols, which usually involved different analgesic treatments such as nonsteroidal anti-inflammatory drugs, opioid drugs, and perioperative regional anaesthesia/analgesia, were becoming more and more popular in recent studies [48]. However, due to the lack of effective data analysis, we cannot reasonably evaluate the effectiveness of these protocols. The treatment of PCA (e.g., fentanyl) is the most involved in our research. Although some of the common side effects of the drug are hypotension, hypertension, bradycardia, tachycardia, hypoxemia, nausea, vomiting, and inhalation, and these adverse effects can be observed during anaesthesia induction [24], we found the incidence of postoperative nausea and vomiting, perioperative transitory hypotension and bradycardia had insignificant differences between the two groups of patients after THA in our meta-analysis (Figure 5). We believe that the results may be influenced by the small sample. Therefore, further studies and analysis must be performed in the future.

Usichenko et al. [21] only conducted a systematic review of AT for postoperative pain and not a meta-analysis due to the low quality and heterogeneity of the included trials. However, new studies that have been conducted in recent years were included, and we conducted various analyses in decreasing the heterogeneity of the results in our meta-analysis. The present evidence of this meta-analysis showed that AT can decrease postoperative VAS pain scores and intraoperative body mass-adjusted fentanyl amount but not the incidence of complications in the patients after THA. Nonetheless, among the available evidence, the GRADE system evaluation results were both at low and extremely low levels, thereby suggesting that we should be cautious about the results of this study.

### 4.1. Study Limitations

Some of the limitations of this study may affect the results, as follows. (1) The sample size of the included studies is small, most studies did not describe the sample size estimation, and most research methods are of low quality. (2) The beginning and end times of AT are unclear and differences in duration and frequency which may be the cause of clinical heterogeneity are significant. (3) Routine analgesia in the control group may also be the cause of clinical heterogeneity. All these factors limited the intensity of the research results. The number of included studies was <10. Hence, funnel plot was not used to analyse publication bias. Therefore, a considerable number of AT-related studies with unified and standardised operating standards and strict design are needed in the future to ensure high level of method quality.

### 4.2. Implications for Future Research and Practice

This review has some implications. First, the main advantages of AT are convenience, safety, and satisfactory postoperative analgesia [49, 50]. Therefore, healthcare workers should be encouraged to learn alternative therapy for postoperative pain. Standardised AT for postoperative pain management should be designed with evidence-based methods, such as the selection and identification of primary and auxiliary acupoints, manual compression guidance and treatment time. Second, patients with chronic pain after the hip operation have extremely high direct costs because of the utilisation of painkillers for years and lengthy rehabilitation programme to ensure the maintenance of patients' motility with sufficient quality of life. Therefore, further studies should pay attention to AT in both acute and chronic postoperative pain and further evaluate the effect of AT as an alternative therapy for pain control after THA. Third, further studies can also include objective evaluation indicators, such as pain effective rate. Other outcome indicators, such as Harris hip score, can be used to evaluate the effect of AT on rehabilitation after THA comprehensively. Most importantly, the methodological quality of future studies must be improved with the explicit descriptions of random sequence generation and allocation concealment which is a reasonable blinding design and an appropriate method for sample size calculation and describe the number and reason of exit in detail.

## 5. Conclusions

The present evidence for the effectiveness of perioperative AT on postoperative pain and intraoperative body mass-adjusted fentanyl amount for the patients after THA was affirmative, but prolongation of the time to first analgesic request and increase in the incidence of complications were not indicated. However, the results of this study still need to be verified by a multicentre, large sample, and high-quality research.

## Figures and Tables

**Table 1:** The baseline characteristics of included trials.

First Author, Year, Setting	Study Design	Participants (n)	Age, Mean(years)	Variety of Disease(n)	Main Outcomes
S^a^1Wetzel, 2011, Germany [23]	Prospective randomized patient-,anesthesiologist-, evaluator-,analyst-blinded, sham-controlled study	Randomized =120; Completed = 116; AT=57; CON= 59;	AT=66(10); CON=67(8)	Degenerative osteoarthritis(116)	Fentanyl amount, adjusted to body mass (mg/kg); Nausea and vomiting; Bradycardia; Transitory hypotension; Time to first analgesic request

S2Usichenko, 2005 Germany [24]	Prospective randomized patient-, anesthesiologist-, evaluator-,analyst-blinded, sham-controlled study	Randomized= 61; Complete =54; AT= 29; CON= 25;	AT=68(10); CON =66(11)	Degenerative osteoarthritis(54)	VAS; Nausea and vomiting; Time to first analgesic request

S3Usichenko, 2006, Germany [25]	Prospective randomized patient-, anesthesiologist-, evaluator-, analyst- blinded, sham-controlled study	Randomized = 64; Completed = 57 AT= 30; CON= 27;	AT=68(9); CON =67(9)	Degenerative osteoarthritis(57)	Fentanyl amount, adjusted to body mass (mg/kg); Bradycardia Transitory hypotension

S4Lv, 2017, China [26]	Randomized controlled trial	Randomized = 98; Completed = 98; AT= 49; CON= 49;	AT=59.8(8.6); CON=59.5(8.7)	Unilateral femoral intertrochanteric fracture or femoral neck fracture(98)	VAS

S5Kong, 2010, China [27]	Randomized controlled trial	Randomized = 60; Completed = 60 AT+C= 30; CON= 30;	NR	NR	VAS

S6Cui, 2016, China [28]	Randomized controlled trial	Randomized = 30; Completed = 30; AT = 15; CON= 15	AT=71(7.8); CON=69(9)	Avascular necrosis of the femoral head(16); Femoral intertrochanteric fracture(5); Femoral neck fracture(9)	VAS

S7Xu, 2014, China [29]	Prospective, randomized, sham-controlled trial	Randomized = 38; Completed = 38 AT= 19; CON=19;	AT=60.7(8.8); CON =59.3(7.7)	Femoral head necrosis(38)	VAS; Nausea and vomiting

S8Shen, 2017, China [30]	Randomized controlled trial	Randomized = 80; Completed = 80; AT= 40; CON= 40	AT=65.8(4.6); CON=66.2,(4.7)	Femoral neck fractures(25); old Femoral neck fractures(5); Femoral head necrosis(24); Hip arthritis(17 ); Rheumatoid arthritis(7); Ankylosing spondylitis (2)	VAS; Time to first analgesic request

S9Tian, 2016, China [31]	Randomized controlled trial	Randomized = 72;Completed = 72; AT = 36; CON=36	NR	Ankylosing spondylitis(1); femoral head necrosis(43); femoral neck fracture( 28)	VAS

Abbreviations: AT=auricular therapy; CON=control group; GA= general anesthesia; AA=auricular acupuncture; APBB=auricular point buried-bean; PCA=patient controlled analgesia; NR=not reported; VAS=visual analogue scale; NR: not reported.

**Table 2:** The detailed treatments in both groups of included trials.

First Author, Year, Setting	Intraoperative Anesthesia	Starting Time of AT	AT Group	Control Group	Details of Sham Acupuncture	Preoperative or Postoperative Analgesics		Details of Conventional Treatments
						NSAIDs	Narcotics	
S^a^1Wetzel, 2011, Germany [23]	GA	Preoperative	*AA+CT*	SA+CT	Three nonacupuncture points	Yes, but no name	NR	Preoperative medication such as NSAIDs, Anticonvulsants, Beta-blocking agents and Antihypertensive agents

S2Usichenko, 2005 Germany [24]	GA	Preoperative	AA+CT	SA + CT	Four nonacupuncture points	Ibuprofen	PCA pump	PCA pump with piritramide in 48h; *Oral Ibuprofen* with 400–800 mg twice a day in 36 hours after the operation

S3Usichenko, 2006, Germany [25]	GA	Preoperative	*AA+CT*	SA+CT	Four nonacupuncture points	NR	NR	NR

S4Lv, 2017, China [26]	NR	Preoperative	APBB+ CT	CT	NR	NR	Opioid analgesics	Postoperative per two hours for the assessment; Opioid analgesics for those sever pain patients; Ice bag cold compress in pain area; other rehabilitation exercise

S6Cui, 2016, China [28]	NR	Postoperative	APBB+ CT	CT	NR	NR	PCA pump	PCA pump in 48 hours; Rehabilitation exercise begin 6 hours after operation

S5Kong, 2010, China [27]	NR	Postoperative	APBB+ CT	CT	NR	NR	PCA pump	PCA pump with fentanyl and Lappaconitine in 48 hours; Rehabilitation exercise: Isometric contraction training of quadriceps femoris after 6 hours and Isometric contraction training of gluteus maximus and gluteus medius after 24 hours; Active motion of knee joint after 2 or 3 days

S7Xu, 2014, China [29]	GA *∗*	Preoperative	APBB+ CT	SA + CT	Nonacupuncture points	Celecoxib	PCA pump	PCA pump with Fentanyl, Tramadol, Tropisetron in 48 hours; *Celecoxib* 200mg was given 3 days before operation, per 12 hours

S8Shen,2017, China [30]	GA	Preoperative	APBB+ CT	CT	NR	NR	PCA pump	PCA pump with sufentanil and tropisetron in 24 hours; health education

S9Tian, 2016,China [31]	GA *∗*	Postoperative	APBB+ CT	CT	NR	NR	PCA pump	PCA pump and rehabilitation guidance for every patients

Abbreviations: AT=auricular therapy; PCA=patient-controlled analgesia; GA= general anesthesia AA=auricular acupuncture; APBB=auricular point buried-bean; *SA=sham acupuncture*; *CT= conventional treatments*; RCT=randomized controlled trial; NSAIDs = nonsteroidal anti-inflammatory drugs; NR= not reported. *∗*information from the authors' email.

**Table 3:** Subgroup analysis for the VAS in the patients after THA by Random-Effect Model.

Variables	Number of points#	Pooled SMD	95%CI	P-value	I-squared	Tau-squared
Total	27	-0.82	(-1.012,-0.642)	<0.001	92.30%	0.2100
the threshold of Observation Time						
24 hours						
<24 hours	14	-1.076	(-1.426,-0.726)	<0.001	82.30%	0.3582
>=24 hours	13	-1.375	(-1.813,-0.938)	<0.001	86.40%	0.5446
48 hours						
<=48 hours	22	-1.174	( -1.496, -0.853)	<0.001	86.10%	0.4969
>48 hours	5	-1.394	(-1.832,-0.955)	0.021	65.30%	0.1593
the Grade of quality						
A	1	0.000	(-0.535,0.535 )	-	-	-
B	4	-1.000	(-1.339,-0.660 )	0.591	0%	0.0000
C	22	-1.311	(-1.62,-1.001)	<0.001	85.70%	0.4583
the type of Intraoperative anesthesia						
GA	18	-1.111	(-1.479,-0.743)	<0.001	86.7%	0.5421
Not reported	9	-1.436	(-1.492,-0.941)	0.002	66.7%	0.1567
the type of control treatment						
SA +CT	5	-1.311	(-1.620,-1.001)	<0.001	85.70%	0.4583
Just CT	22	-0.780	(-1.270,-0.290)	0.022	65.10%	0.2022
General Analgesics (NASIDs)						
Yes	5	-0.772	(-0.172,-0.292)	0.001	65.8%	0.2045
No	22	-1.217	(-1.492,-0.941)	0.002	85.2%	0.4583
the starting time of AT						
Preoperative	14	-1.241	(-1.071,-1.207 )	<0.001	86.2%	0.2922
Postoperative	13	-1.114	(-1.221,-1.008)	<0.001	83.5%	0.5877

#: The number of points was including the different observation time for the patients after THA. SA=sham acupuncture; CT= conventional treatments; GA= general anesthesia; SMD= standardised mean difference; CI= confidence interval.

**Table 4:** The GRADE tool for the pooled results of different period in the patients after total hip arthroplasty.

Outcomes	No. of studies	Quality assessment					Summary of results				Importance
		Risk of Bias	Inconsistency	Indirectness	Imprecision	Publication Bias	No. of patients		95%CI	Quality	
							Auricular therapy	Control			
*Postoperative-results*											
Pain intensity (VAS-10)											
At 6h	2	Serious^(1)^	Serious^(2)^	no	very serious^(4)(5)^	no	55	55	SMD -0.74 (-1.80,0.32)	VERY LOW	CRITICAL
At 12h	6	serious^(1)^	serious^(2)^	no	serious^(4)^	no	189	189	SMD -1.03 (-1.51,-0.55)	VERY LOW	CRITICAL
At 24h	6	serious^(1)^	serious^(2)^	no	Serious^(4)^	no	189	189	SMD -0.95 (-1.53,-0.37)	VERY LOW	CRITICAL
At 36h	3	serious^(1)^	serious^(2)^	no	very serious^(4)(5)^	no	93	89	SMD -0.39 (-0.83,0.05)	VERY LOW	CRITICAL
At 48h	5	serious^(1)^	serious^(2)^	no	serious^(4)^	no	149	149	SMD -0.89 (-1.48,-0.30)	VERY LOW	CRITICAL
At 72h	3	serious^(1)^	no	no	serious^(4)^	no	94	94	SMD -0.79 (-0.92,-0.66)	LOW	CRITICAL
At 5d	1	serious^(1)^	no	no	serious^(4)^	no	30	30	SMD -0.60 (-0.94,-0.26)	LOW	CRITICAL
At 7d	1	serious^(1)^	no	no	serious^(4)^	no	30	30	SMD -0.68 (-1.01,-0.35)	LOW	CRITICAL
Time to first analgesic request (min)	3	no	serious^(2)^	serious^(3)^	very serious^(4)(5)^	no	126	124	SMD20.95. (-11.01,52.9)	VERY LOW	IMPORTANT
*Intraoperative results*											
Fentanyl amount, adjusted to body mass (*μ*g/kg)	2	no	no	serious^(3)^	serious^(4)^	no	87	86	SMD -0.73 (-1.09,-0.36)	LOW	IMPORTANT
*Perioperative complications*											
Nausea and vomiting	3	no	no	serious^(3)^	very serious^(4)(5)^	no	19/105	23/103	OR 0.72 (0.36,1.46)	VERY LOW	IMPORTANT
Bradycardia	2	no	no	serious^(3)^	very serious^(4)(5)^	no	14/87	12/86	OR 1.18 (0.51,2.72)	VERY LOW	IMPORTANT
Transitory hypotension	2	no	no	serious^(3)^	very serious^(4)(5)^	no	36/90	35/90	OR 1.06 (0.58,1.92)	VERY LOW	IMPORTANT

Note: (1) allocation sequence concealment and blinding are missing, (2) I^2^>50%, *P*<0.1, (3) indirectness, (4) insufficient sample size, (5) confidence interval spanning invalid lines.

## Supplementary Materials

Supplementary Materials This section contains a detailed description of search strategies including the databases of PubMed, Excerpta Medica Database (Embase), Cochrane Central Register of Controlled Trials, Web of Science, Science Direct, PsycINFO, Cumulative Index to Nursing and Allied Health Literature, and Allied and Complementary Medicine. All search strategies are based on PICOS principles.
