# Supplementary material for: Effectiveness of Perioperative Auricular Therapy on Postoperative Pain after Total Hip Arthroplasty: A Systematic Review and Meta-Analysis of Randomised Controlled Trials
Source: Evid Based Complement Alternat Med. 2019 Mar 3;2019:2979780. doi: 10.1155/2019/2979780 (PMC6420993; doi:10.1155/2019/2979780)
Supplement: Supplementary Materials — This section contains a detailed description of search strategies including the databases of PubMed, Excerpta Medica Database (Embase), Cochrane Central Register of Controlled Trials, Web of Science, Science Direct, PsycINFO, Cumulative Index to Nursing and Allied Health Literature, and Allied and Complementary Medicine. All search strategies are based on PICOS principles. [file 2979780.f1.docx]

**Supplemental materials for Effectiveness of Perioperative Auricular Therapy on Postoperative Pain after Total Hip Arthroplasty –A Systematic Review and Meta-analysis of Randomized Controlled Trails**

**Contents**

[Search strategy of all the databases 3](#_Toc536131271)

[Proposed Databases (8 DataBases) 3](#_Toc536131272)

[PubMed 2018-04-16 (8 items) 3](#_Toc536131273)

[EMBase 2018-04-15 ( 8 items) 4](#_Toc536131274)

[Cochrane Library (Cochrane Central Register of Controlled Trials, CENTRAL)2018-04-16 ( 7items) 8](#_Toc536131275)

[Science Direct 2018-08-17 (15 items) 9](#_Toc536131276)

[PsycINFO 2018-08-17 (1 items) 9](#_Toc536131277)

[Allied and Complementary Medicine (AMED) 2018-08-17 (2 item) 11](#_Toc536131278)

# Search strategy of all the databases

## Proposed Databases (8 DataBases)

PubMed

EMBase

Cochrane Library (Cochrane Central Register of Controlled Trials, CENTRAL)

Web of Science

Science Direct

PsycINFO

Cumulative Index to Nursing and Allied Health Literature (CINAHL)

Allied and Complementary Medicine (AMED)

**2) Mesh Terms and Key Words**

**① English**

**[Mesh]** Auriculotherapy; Acupuncture, ear

**[Entry Term and Free Words]**

| Ear + Acupunctur* | Auricular + Acupunctur* | Auricular + Acupressur* |
| --- | --- | --- |
| Auriculotherap* | Auricular + Poin* | Auricular + Plaste* |
| Ear + Poin* | Ear + Acupoin* | Ear + Acupressur* |
| Otopoin* | Auricular + Massag* | Ear + Massag* |
| Ear Hol* | Vaccaria* | Seed* |
| Magne*+ Auricu* | Magne*+ Ear | Erxue |

**[Mesh] Pain, Postoperative**

**[Entry Term and Free Words] Postoperative Pain*;*pain;pain*;Ache*;Suffering*;*Suffering**

**[Mesh] Arthroplasty, Replacement, Hip**

**[Entry Term and Free Words]**

| Total hip arthroplasty | Total hip replacement* | THR |
| --- | --- | --- |
| Arthroplasty, Hip Replacement | Arthroplasties, Replacement, Hip | Hip Prosthesis Implantation* |
| Implantation*, Hip Prosthesis | Prosthesis Implantation*, Hip | Hip Replacement Arthroplasty |
| Replacement Arthroplast*, Hip | Arthroplasties, Hip Replacement | Hip Replacement Arthroplasties |
| Hip Replacement*, Total | Replacement*, Total Hip |  |

**3) Searching Strategies**

## PubMed 2018-04-16 (8 items)

**#1** "auriculotherapy"[MeSH Terms] OR "acupuncture, ear"[MeSH Terms] **393**

**#2** ((((((((((((((((((auriculotherap*[Title/Abstract]) OR (acupunctur*[Title/Abstract] AND ear*[Title/Abstract])) OR (acupunctur*[Title/Abstract] AND auricu*[Title/Abstract])) OR (acupressur*[Title/Abstract] AND ear*[Title/Abstract])) OR (acupressur*[Title/Abstract] AND auricu*[Title/Abstract])) OR (auricu*[Title/Abstract] AND poin*[Title/Abstract])) OR (ear[Title/Abstract] AND poin*[Title/Abstract])) OR (ear[Title/Abstract] AND acupoin*[Title/Abstract])) OR (auricu*[Title/Abstract] AND plaster*[Title/Abstract])) OR (massag*[Title/Abstract] AND ear*[Title/Abstract])) OR (ear[Title/Abstract] AND plaster*[Title/Abstract])) OR (massag*[Title/Abstract] AND auricu*[Title/Abstract])) OR (magne*[Title/Abstract] AND ear*[Title/Abstract])) OR (magne*[Title/Abstract] AND auricu*[Title/Abstract])) OR otopoin*[Title/Abstract]) OR (ear[Title/Abstract] AND hol*[Title/Abstract])) OR vaccaria*[Title/Abstract]) OR seed*[Title/Abstract]) OR erxue[Title/Abstract] **186721**

**#3 #1 OR #2** **186788**

#4 "Pain, Postoperative"[Mesh] **35443**

#5 (((((Postoperative Pain*[Title/Abstract]) OR *pain[Title/Abstract]) OR pain*[Title/Abstract]) OR Ache*[Title/Abstract]) OR Suffering*[Title/Abstract]) OR *Suffering[Title/Abstract] **724087**

**#6 #4 OR #5** **732050**

**#7** "Arthroplasty, Replacement, Hip"[Mesh] **22698**

**#8**  (((((((((((((Total hip arthroplasty[Title/Abstract]) OR Total hip replacement*[Title/Abstract]) OR THR[Title/Abstract]) OR Arthroplasty, Hip Replacement[Title/Abstract]) OR Arthroplasties, Replacement, Hip[Title/Abstract]) OR Hip Prosthesis Implantation*[Title/Abstract]) OR Implantation*, Hip Prosthesis[Title/Abstract]) OR Prosthesis Implantation*, Hip[Title/Abstract]) OR Hip Replacement Arthroplasty[Title/Abstract]) OR Replacement Arthroplast*, Hip[Title/Abstract]) OR Arthroplasties, Hip Replacement[Title/Abstract]) OR Hip Replacement Arthroplasties[Title/Abstract]) OR Hip Replacement*, Total[Title/Abstract]) OR Replacement*, Total Hip[Title/Abstract] **20343**

**#9** **#7 OR #8** **29115**

**#10 #3 AND #6 AND #9** **32**

**#11** (((((((("randomized controlled trial"[Publication Type]) OR "controlled clinical trial"[Publication Type]) OR "randomized"[Title/Abstract]) OR "randomised"[Title/Abstract]) OR "placebo"[Title/Abstract]) OR "sham"[Title/Abstract]) OR "randomly"[Title/Abstract]) OR "trial"[Title/Abstract]) OR "groups"[Title/Abstract] **2698644**

**#12** (animals[MeSH Terms] NOT (humans[MeSH Terms] AND animals[MeSH Terms])) **4442594**

**#13 #11 NOT #12 2265821**

**#14** **#10 AND #13** **8**

## EMBase 2018-04-15 ( 8 items)

No. Query Results Results Date

#79. #47 AND #78  **8** 15 Apr 2018

#78. #70 NOT #77 1,838,588 15 Apr 2018

#77. #74 NOT #76 6,443,968 15 Apr 2018

#76. #74 AND #75 19,334,768 15 Apr 2018

#75. 'human'/exp 19,334,768 15 Apr 2018

#74. #71 OR #72 OR #73 25,778,736 15 Apr 2018

#73. 'animal experiment'/exp 2,198,558 15 Apr 2018

#72. 'nonhuman'/exp 5,385,028 15 Apr 2018

#71. 'animal'/exp 24,358,746 15 Apr 2018

#70. #68 OR #69 2,080,366 15 Apr 2018

#69. #52 OR #53 OR #54 OR #55 OR #56 OR #57 OR #58 OR 1,862,279 15 Apr 2018

#59 OR #60 OR #61 OR #62 OR #63 OR #64 OR #65 OR

#66 OR #67

#68. #48 OR #49 OR #50 OR #51 706,182 15 Apr 2018

#67. (trebl* NEXT/3 blind*):ab,ti 15 Apr 2018

#66. (trebl* NEXT/3 mask*):ab,ti 15 Apr 2018

#65. (singl* NEXT/3 mask*):ab,ti 674 15 Apr 2018

#64. (singl* NEXT/3 blind*):ab,ti 22,773 15 Apr 2018

#63. (doubl* NEXT/3 mask*):ab,ti 3,639 15 Apr 2018

#62. (doubl* NEXT/3 blind*):ab,ti 187,350 15 Apr 2018

#61. (cross NEXT/3 over*):ab,ti 30,052 15 Apr 2018

#60. 'placebo':ab,ti 267,648 15 Apr 2018

#59. (tripl* NEXT/3 mask*):ab,ti 76 15 Apr 2018

#58. (tripl* NEXT/3 blind*):ab,ti 814 15 Apr 2018

#57. 'volunteer*':ab,ti 230,520 15 Apr 2018

#56. 'allocat*':ab,ti 125,004 15 Apr 2018

#55. 'assign*':ab,ti 332,680 15 Apr 2018

#54. 'placebo':ab,ti 267,648 15 Apr 2018

#53. 'crossover*':ab,ti 65,179 15 Apr 2018

#52. 'random*':ab,ti 1,278,598 15 Apr 2018

#51. 'crossover procedure'/exp 54,560 15 Apr 2018

#50. 'double blind procedure'/exp 147,713 15 Apr 2018

#49. 'single blind procedure'/exp 30,977 15 Apr 2018

#48. 'controlled clinical trial'/exp 659,513 15 Apr 2018

#47. #26 AND #33 AND #46 33 15 Apr 2018

#46. #34 OR #45 55,175 15 Apr 2018

#45. #35 OR #36 OR #37 OR #38 OR #39 OR #40 OR #41 OR 53,874 15 Apr 2018

#42 OR #43 OR #44

#44. (replacement* NEAR/3 hip NEAR/3 total):ti,ab 12,039 15 Apr 2018

#43. (replacement NEAR/3 hip NEAR/3 326 15 Apr 2018

arthroplast*):ti,ab

#42. (implantation* NEAR/3 hip NEAR/3 79 15 Apr 2018

prosthesis):ti,ab

#41. (arthroplasty NEAR/3 hip NEAR/3 326 15 Apr 2018

replacement):ti,ab

#40. 'hip replacement arthroplasties':ti,ab 16 15 Apr 2018

#39. 'hip replacement arthroplasty':ti,ab 128 15 Apr 2018

#38. 'hip prosthesis implantation*':ti,ab 28 15 Apr 2018

#37. 'thr':ti,ab 28,511 15 Apr 2018

#36. 'total hip replacement*':ti,ab 10,634 15 Apr 2018

#35. 'total hip arthroplasty':ti,ab 17,090 15 Apr 2018

#34. 'hip replacement'/exp 2,016 15 Apr 2018

#33. #27 OR #32 1,048,017 15 Apr 2018

#32. #28 OR #29 OR #30 OR #31 1,033,041 15 Apr 2018

#31. 'suffering*':ab,ti 176,637 15 Apr 2018

#30. 'ache*':ab,ti 23,172 15 Apr 2018

#29. 'pain*':ab,ti 857,700 15 Apr 2018

#28. 'postoperative pain*':ab,ti 29,158 15 Apr 2018

#27. 'postoperative pain'/exp 57,879 15 Apr 2018

#26. #1 OR #25 53,992 15 Apr 2018

#25. #2 OR #3 OR #4 OR #5 OR #6 OR #7 OR #8 OR #9 OR 13,765 15 Apr 2018

#10 OR #11 OR #12 OR #13 OR #14 OR #15 OR #16 OR

#17 OR #18 OR #19 OR #20 OR #21 OR #22 OR #23 OR

#24

#24. 'erxue*':ab,ti 15 Apr 2018

#23. 'earhole*':ab,ti 7 15 Apr 2018

#22. 'otopoin*':ab,ti 20 15 Apr 2018

#21. 'auricular plaster':ab,ti 27 15 Apr 2018

#20. (magne* NEAR/15 auricu*):ti,ab 78 15 Apr 2018

#19. (magne* NEAR/15 ear*):ti,ab 11,838 15 Apr 2018

#18. (seed* NEAR/15 ear):ti,ab 185 15 Apr 2018

#17. (seed* NEAR/15 auricu*):ti,ab 130 15 Apr 2018

#16. (cowherb NEAR/15 auricu*):ti,ab 4 15 Apr 2018

#15. (cowherb NEAR/15 ear*):ti,ab 3 15 Apr 2018

#14. (massag* NEAR/3 ear*):ti,ab 59 15 Apr 2018

#13. (massag* NEAR/3 auricu*):ti,ab 7 15 Apr 2018

#12. (vaccaria* NEAR/15 auricu*):ti,ab 32 15 Apr 2018

#11. (vaccaria* NEAR/15 ear*):ti,ab 13 15 Apr 2018

#10. (ear NEAR/3 acupoint*):ti,ab 45 15 Apr 2018

#9. (ear NEAR/3 poin*):ti,ab 291 15 Apr 2018

#8. (ear NEAR/3 plaster*):ti,ab 9 15 Apr 2018

#7. (auricu* NEAR/3 poin*):ti,ab 342 15 Apr 2018

#6. (auricu* NEAR/3 acupressur*):ti,ab 136 15 Apr 2018

#5. (ear NEAR/3 acupressur*):ti,ab 30 15 Apr 2018

#4. (auricu* NEAR/3 acupunctur*):ti,ab 625 15 Apr 2018

#3. (ear NEAR/3 acupuncture*):ti,ab 365 15 Apr 2018

#2. 'auriculotherap*':ab,ti 178 15 Apr 2018

#1. 'acupuncture'/exp 41,605 15 Apr 2018

## Cochrane Library (Cochrane Central Register of Controlled Trials, CENTRAL)2018-04-16 ( 7items)

**
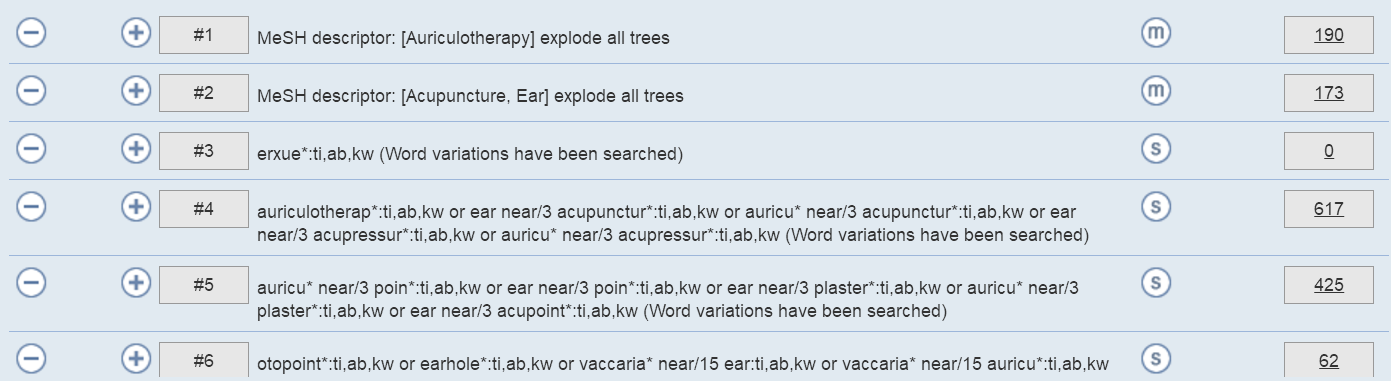
**

**
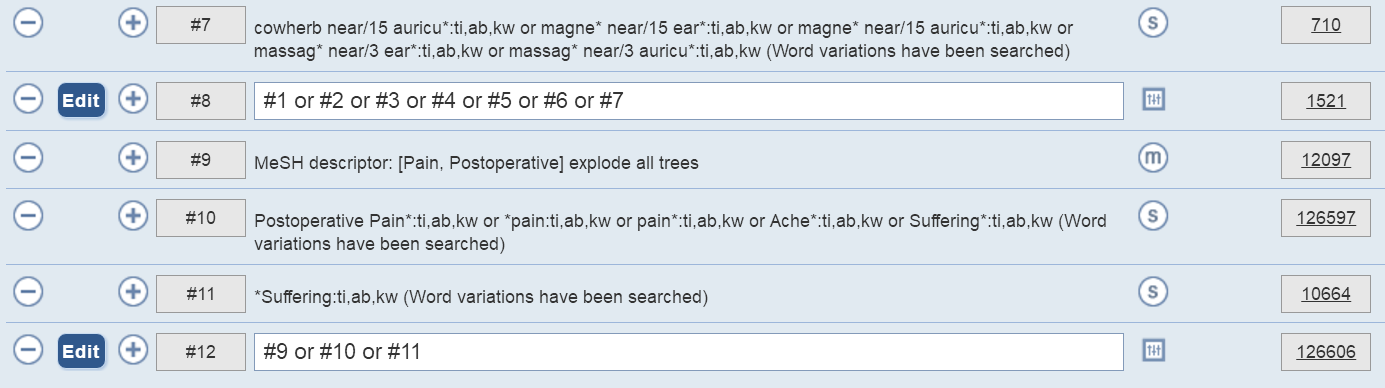
**

**
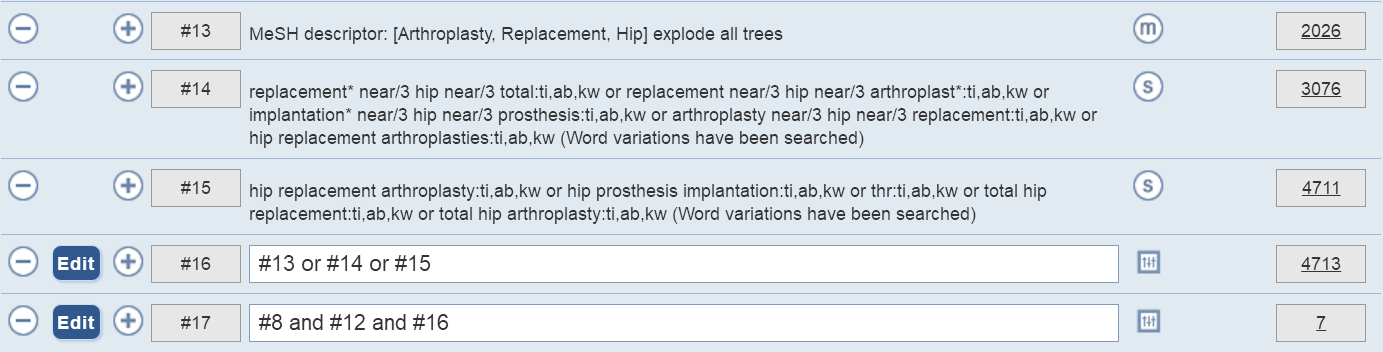
**

Web of Science 2018-08-16 (11 items)

#1 TOPIC: (auriculotherap* OR (ear NEAR/3 acupunctur*) OR (auricu* NEAR/3 acupunctur*) OR (ear NEAR/3 acupressur*) OR (auricu* NEAR/3 acupressur*) OR (auricu* NEAR/3 poin*) OR (ear NEAR/3 plaster*) OR (auricu* NEAR/3 plaster*) OR (ear NEAR/3 poin*) OR (ear NEAR/3 acupoint*) OR otopoint* OR earhole* OR (vaccaria* NEAR/15 ear*) OR (vaccaria* NEAR/15 auricu*) OR (cowherb NEAR/15 ear*) OR (cowherb NEAR/15 auricu*) OR (magne* NEAR/15 ear*) OR (magne* NEAR/15 auricu*) OR (massag* NEAR/3 ear*) OR (massag* NEAR/3 auricu*) OR erxue*) 130539

#2 TOPIC: (postoperative pain) or (postoperative pain*) or (pain*) or (ache*) or (suffering*) or (*suffering) 1769252

#3 TOPIC: (hip replacement) or ( arthroplasty,replacement,hip) or (total hip arthroplasty) or (total hip replacement) or (thr) or (hip prosthesis implantation*) or (hip replacement arthroplasty) or (hip replacement arthroplasties) or (arthroplasty NEAR/3 hip NEAR/3 replacement) or (implantation* NEAR/3 hip NEAR/3 prosthesis) or (replacement NEAR/3 hip NEAR/3 arthroplast*) or (replacement* NEAR/3 hip NEAR/3 total)

123023

**#4 #1 AND #2 AND #3 27**

**#5** TOPIC: (random* OR crossover* OR (cross NEAR/3 over*) OR placebo OR (doubl* NEAR/3 blind*) OR (doubl* NEAR/3 mask*) OR (singl* NEAR/3 blind*) OR (singl* NEAR/3 mask*) OR (trebl* NEAR/3 blind*) OR (trebl* NEAR/3 mask*) OR (tripl* NEAR/3 blind*) OR (tripl* NEAR/3 mask*) OR assign* OR allocat* OR volunteer*) Indexes=SCI-EXPANDED, SSCI, A&HCI, CPCI-S, CPCI-SSH Timespan=All years **4756166**

**#6 #4 AND #5** **11**

## Science Direct 2018-08-17 (15 items)

**#1** “auriculotherapy” OR “ear acupuncture” **755**

**#2** “postoperative pain” OR “pain” **1,162,518**

**#3** “hip replacement” OR “hip arthroplasty” OR “thr” OR “hip prosthesis implantation” **163,523**

**#4 #1 AND #2 AND #3** **15**

## PsycINFO 2018-08-17 (1 items)

**#1** SU"Acupuncture" **1,557**

**#2** MJ "Acupuncture" **1,229**

**#3** TI auriculotherap* OR (ear N3 acupunctur*) OR (auricu* N3 acupunctur*) OR (ear N3 acupressur*) OR (auricu* N3 acupressur*) OR (auricu* N3 poin*) OR (ear N3 poin*) OR (ear N3 plaster*) OR (auricu* N3 plaster*) OR (ear N5 acupoint*) OR otopoint* OR earhole* OR (vaccaria* N15 ear) OR (vaccaria* N15 auricu*) OR (cowherb N15 ear*) OR (cowherb N15 auricu*) OR (magne* N15 ear*) OR (magne* N15 auricu*) OR (massag* N3 ear*) OR (massag* N3 auricu*) or erxue*  **263**

**#4** AB auriculotherap* OR (ear N3 acupunctur*) OR (auricu* N3 acupunctur*) OR (ear N3 acupressur*) OR (auricu* N3 acupressur*) OR (auricu* N3 poin*) OR (ear N3 poin*) OR (ear N3 plaster*) OR (auricu* N3 plaster*) OR (ear N5 acupoint*) OR otopoint* OR earhole* OR (vaccaria* N15 ear) OR (vaccaria* N15 auricu*) OR (cowherb N15 ear*) OR (cowherb N15 auricu*) OR (magne* N15 ear*) OR (magne* N15 auricu*) OR (massag* N3 ear*) OR (massag* N3 auricu*) or erxue* **1579**

**#5 #1 OR #2 OR #3 OR #4** **3174**

**#6** SU  Pain, Postoperative  **1474**

**#7** MJ  Pain, Postoperative  **107,919**

**#8** TI (postoperative pain) or (postoperative pain*) or (pain*) or (ache*) or (suffering*) or (*suffering) **43,117**

**#9** AB(postoperative pain) or (postoperative pain*) or (pain*) or (ache*) or (suffering*) or (*suffering) **135,797**

**#10 #6 OR #7 OR #8 OR #9 139,849**

**#11**SU hip replacement **276**

**#12** MJ hip replacement  **17787**

**#13** SU Arthroplasty, Replacement, Hip **212**

**#14** MJ Arthroplasty, Replacement, Hip **17869**

**#15** TI(hip replacement) or ( arthroplasty,replacement,hip) or (total hip arthroplasty) or (total hip replacement) or (thr) or (hip prosthesis implantation*) or (hip replacement arthroplasty) or (hip replacement arthroplasties) or (arthroplasty NEAR/3 hip NEAR/3 replacement) or (implantation* NEAR/3 hip NEAR/3 prosthesis) or (replacement NEAR/3 hip NEAR/3 arthroplast*) or (replacement* NEAR/3 hip NEAR/3 total) **178**

**#16** AB(hip replacement) or ( arthroplasty,replacement,hip) or (total hip arthroplasty) or (total hip replacement) or (thr) or (hip prosthesis implantation*) or (hip replacement arthroplasty) or (hip replacement arthroplasties) or (arthroplasty NEAR/3 hip NEAR/3 replacement) or (implantation* NEAR/3 hip NEAR/3 prosthesis) or (replacement NEAR/3 hip NEAR/3 arthroplast*) or (replacement* NEAR/3 hip NEAR/3 total) **576**

**#17 #11 OR #12 OR #13 OR #14 OR #15 OR #16** **674**

**#18 #5 AND #10 AND #17** **2**

**#19** TI(random* OR crossover* OR cross NEAR/3 over* OR placebo OR double* NEAR/3 blind* OR double* NEAR/3 mask* OR single* NEAR/3 blind* OR single* NEAR/3 mask* OR treble* NEAR/3 blind* OR treble* NEAR/3 mask* OR triple* NEAR/3 blind* OR triple* NEAR/3 mask* OR assign* OR allocate* OR volunteer*) **38717**

**#20** AB(random* OR crossover* OR cross NEAR/3 over* OR placebo OR double* NEAR/3 blind* OR double* NEAR/3 mask* OR single* NEAR/3 blind* OR single* NEAR/3 mask* OR treble* NEAR/3 blind* OR treble* NEAR/3 mask* OR triple* NEAR/3 blind* OR triple* NEAR/3 mask* OR assign* OR allocate* OR volunteer*) **284427**

**#21 #19 OR #20** **288266**

**#22 #18 AND #21** **1**

**Cumulative Index to Nursing and Allied Health Literature** (CINAHL) **2018-08-17 (2 items)**
**#1** (MM "Auriculotherapy+") **224**

**#2** (MM "Acupuncture, Ear") **175**

**#3** TI auriculotherap* OR (ear N3 acupunctur*) OR (auricu* N3 acupunctur*) OR (ear N3 acupressur*) OR (auricu* N3 acupressur*) OR (auricu* N3 poin*) OR (ear N3 poin*) OR (ear N3 plaster*) OR (auricu* N3 plaster*) OR (ear N5 acupoint*) OR otopoint* OR earhole* OR (vaccaria* N15 ear) OR (vaccaria* N15 auricu*) OR (cowherb N15 ear*) OR (cowherb N15 auricu*) OR (magne* N15 ear*) OR (magne* N15 auricu*) OR (massag* N3 ear*) OR (massag* N3 auricu*) or erxue* **900**

**#4** AB auriculotherap* OR (ear N3 acupunctur*) OR (auricu* N3 acupunctur*) OR (ear N3 acupressur*) OR (auricu* N3 acupressur*) OR (auricu* N3 poin*) OR (ear N3 poin*) OR (ear N3 plaster*) OR (auricu* N3 plaster*) OR (ear N5 acupoint*) OR otopoint* OR earhole* OR (vaccaria* N15 ear) OR (vaccaria* N15 auricu*) OR (cowherb N15 ear*) OR (cowherb N15 auricu*) OR (magne* N15 ear*) OR (magne* N15 auricu*) OR (massag* N3 ear*) OR (massag* N3 auricu*) or erxue* **1910**

**#5 #1 OR #2 OR #3 OR #4 2508**

**#6** (MM "Postoperative Pain ") **9478**

**#7** TI (postoperative pain) or (postoperative pain*) or (pain*) or (ache*) or (suffering*) or (*suffering) **103,944**

**#8** AB (postoperative pain) or (postoperative pain*) or (pain*) or (ache*) or (suffering*) or (*suffering) **169,284**

**#9 #6 OR #7 OR #8 227,017**

**#10** (MM "Arthroplasty, Replacement, Hip ") **12,055**

**#11** TI (hip replacement) or ( arthroplasty,replacement,hip) or (total hip arthroplasty) or (total hip replacement) or (thr) or (hip prosthesis implantation*) or (hip replacement arthroplasty) or (hip replacement arthroplasties) or (arthroplasty NEAR/3 hip NEAR/3 replacement) or (implantation* NEAR/3 hip NEAR/3 prosthesis) or (replacement NEAR/3 hip NEAR/3 arthroplast*) or (replacement* NEAR/3 hip NEAR/3 total)  **6758**

**#12** AB (hip replacement) or ( arthroplasty,replacement,hip) or (total hip arthroplasty) or (total hip replacement) or (thr) or (hip prosthesis implantation*) or (hip replacement arthroplasty) or (hip replacement arthroplasties) or (arthroplasty NEAR/3 hip NEAR/3 replacement) or (implantation* NEAR/3 hip NEAR/3 prosthesis) or (replacement NEAR/3 hip NEAR/3 arthroplast*) or (replacement* NEAR/3 hip NEAR/3 total)  **8409**

**#13 #10 OR #11 OR #12** **194,859**

**#14 #5 AND #9 AND #13** **5**

**#15** (MM "Quantitative Studies+") **410**

**#16** (MM "Clinical Trials+") OR (MM "Randomized Controlled Trials") **17601**

**#17** (MM "Random Assignment") **199**

**#18** (MM "Placebos") **1025**

**#19** TI random* OR crossover* OR (cross N3 over*) OR placebo OR (doubl* N3 blind*) OR (doubl* N3 mask*) OR (singl* N3 blind*) OR (singl* N3 mask*) OR (trebl* N3 blind*) OR (trebl* N3 mask*) OR (tripl* N3blind*) OR (tripl* N3 mask*) OR assign* OR allocat* OR volunteer* **101,303**

**#20** AB random* OR crossover* OR (cross N3 over*) OR placebo OR (doubl* N3 blind*) OR (doubl* N3 mask*) OR (singl* N3 blind*) OR (singl* N3 mask*) OR (trebl* N3 blind*) OR (trebl* N3 mask*) OR (tripl* N3blind*) OR (tripl* N3 mask*) OR assign* OR allocat* OR volunteer* **301,413**

**#21 #15 OR #16 OR #17 OR #18 OR #19 OR #20** **354,435**

**#22 #14 AND #21** **2**

## Allied and Complementary Medicine (AMED) 2018-08-17 (2 item)

**#1** Ear acupuncture.sh. **433**

**#2**("auriculotherap*" or "ear adj3 acupunctur*" or "auricu* adj3 acupunctur*" or "ear adj3 acupressur*" or "auricu* adj3 acupressur*" or "auricu* adj3 poin*" or "auricular plaster" or "ear adj3 plaster*" or "ear adj3 poin*" or "ear adj3 acupoint*" or "otopoin*" or "earhole*" or "vaccaria* adj15 ear*" or "vaccaria* adj15 auricu*" or "massag* adj3 auricu*" or "massag* adj3 ear*" or "cowherb adj15 ear*" or "cowherb adj15 auricu*" or "seed* adj15 auricu*" or "seed* adj15 ear" or "magne* adj15 ear*" or "magne* adj15 auricu*" or "erxue*").ti. **56**

**#3** ("auriculotherap*" or "ear adj3 acupunctur*" or "auricu* adj3 acupunctur*" or "ear adj3 acupressur*" or "auricu* adj3 acupressur*" or "auricu* adj3 poin*" or "auricular plaster" or "ear adj3 plaster*" or "ear adj3 poin*" or "ear adj3 acupoint*" or "otopoin*" or "earhole*" or "vaccaria* adj15 ear*" or "vaccaria* adj15 auricu*" or "massag* adj3 auricu*" or "massag* adj3 ear*" or "cowherb adj15 ear*" or "cowherb adj15 auricu*" or "seed* adj15 auricu*" or "seed* adj15 ear" or "magne* adj15 ear*" or "magne* adj15 auricu*" or "erxue*").ab. **43**

**#4 #1 OR #2 OR #3 462**

**#5** Pain, Postoperative.sh. **298**

**#6**("Postoperative Pain*" or "*pain" or "pain*" or "Ache*" or "Suffering*" or "*Suffering").ti. **14502**

**#7** ("Postoperative Pain*" or "*pain" or "pain*" or "Ache*" or "Suffering*" or "*Suffering").ab. **23256**

**#8 #5 OR #6 OR #7 29687**

**#9** Arthroplasty, Replacement, Hip.sh. **440**

**#10** ("Total hip arthroplasty" or "Total hip replacement*" or "THR" or "Arthroplasty, Hip Replacement" or "Arthroplasties, Replacement, Hip" or "Hip Prosthesis Implantation*" or "Implantation*, Hip Prosthesis" or "Prosthesis Implantation*, Hip" or "Hip Replacement Arthroplasty" or "Replacement Arthroplast*, Hip" or "Arthroplasties, Hip Replacement" or "Hip Replacement Arthroplasties" or "Hip Replacement*, Total" or "Replacement*, Total Hip").ti. **387**

**#11**("Total hip arthroplasty" or "Total hip replacement*" or "THR" or "Arthroplasty, Hip Replacement" or "Arthroplasties, Replacement, Hip" or "Hip Prosthesis Implantation*" or "Implantation*, Hip Prosthesis" or "Prosthesis Implantation*, Hip" or "Hip Replacement Arthroplasty" or "Replacement Arthroplast*, Hip" or "Arthroplasties, Hip Replacement" or "Hip Replacement Arthroplasties" or "Hip Replacement*, Total" or "Replacement*, Total Hip").ab. **468**

**#12 #9 OR #10 OR #11** **787**

**#13 #4 AND #8 AND #12** **2**

**#15** (Random allocation or Clinical trials or Randomized controlled trials or Placebos).sh. **4304**

**#16** ("random*" or "crossover*" or "cross adj3 over*" or "placebo" or "doubl* adj3 blind*" or "doubl* adj3 mask*" or "singl* adj3 blind*" or "singl* adj3 mask*" or "trebl* adj3 blind*" or "trebl* adj3 mask*" or "tripl* adj3 blind*" or "allocat*" or " volunteer*" or "tripl* adj3 mask*" or "assign*").ti. **6467**

**#17** ("random*" or "crossover*" or "cross adj3 over*" or "placebo" or "doubl* adj3 blind*" or "doubl* adj3 mask*" or "singl* adj3 blind*" or "singl* adj3 mask*" or "trebl* adj3 blind*" or "trebl* adj3 mask*" or "tripl* adj3 blind*" or "allocat*" or " volunteer*" or "tripl* adj3 mask*" or "assign*").ab. **21148**

**#18 #15 OR #16 OR #17** **25145**

**#19 #14 AND #18** **2**
